# In Praise of Quantum Uncertainty

**DOI:** 10.3390/e22030302

**Published:** 2020-03-06

**Authors:** Eliahu Cohen, Avishy Carmi

**Affiliations:** 1 Faculty of Engineering & the Institute of Nanotechnology and Advanced Materials, Bar Ilan University, Ramat Gan 5290002, Israel; 2 Center for Quantum Information Science and Technology & Faculty of Engineering Sciences, Ben-Gurion University of the Negev, Beersheba 8410501, Israel; avcarmi@bgu.ac.il

**Keywords:** quantum mechanics, uncertainty, indeterminism, quantum nonlocality, entanglement, contextuality

## Abstract

Quantum uncertainty has a tremendous explanatory power. Coherent superposition, quantum equations of motion, entanglement, nonlocal correlations, dynamical nonlocality, contextuality, discord, counterfactual protocols, weak measurements, quantization itself, and even preservation of causality can be traced back to quantum uncertainty. We revisit and extend our previous works, as well as some other works of the community, in order to account for the above claims. Special emphasis is given to the connection between uncertainty and nonlocality, two notions which evolved quite independently and may seem distinct but, in fact, are tightly related. Indeterminism, or more precisely, locally consistent indeterminism, should be understood as the enabler of most quantum phenomena (and possibly all of them).

## 1. Introduction

Classical mechanics and classical field theories (including electromagnetism) are deterministic. Full specification of the initial conditions allows, in principle, the determination of the state of the system at any later moment. It is also possible to sharply measure all the physical variables of the system at will. Quantum indeterminism may, therefore, appear as a severe limitation of quantum mechanics. Perhaps we would have liked to know simultaneously all physical variables of a microscopic quantum system, but alas we cannot. Is that a curse? No, it is actually a blessing. As ironically indicated by George Orwell, “ignorance is strength”, or at least it is in quantum mechanics.

How is this so? That is exactly the question we wish to answer here, based on a substantial amount of evidence provided in our previous works and in the works of additional authors.

Before we do that, we need to define better what we mean by indeterminism. We would like to have an empirical notion which is not bounded to a specific theoretical model. Following Ref. [1], we shall henceforth ascribe the property of indeterminism to any system having at least two physical variables which cannot be jointly measured with absolute precision. In other words, within our framework indeterminism amounts to the existence of random variables $A_{0}$, $A_{1}$ and a non-zero complex number *r*, such that $\Delta_{A_{1}}^{2}\Delta_{A_{0}}^{2}\geq {\left|r\right|}^{2}$. The variance $\Delta_{A_{i}}^{2}=E\left[A_{i}^{2}\right]-E{\left[A_{i}\right]}^{2}$ can be measured in a sequence of experiments, given that the average converges to the expected value (denoted here by *E*). In the following, it will be constructive to encode the above inequality in a positive semi-definiteness condition:

(1) $$\left[\begin{array}{cc} \Delta_{A_{1}}^{2} & r\\ r^{*} & \Delta_{A_{0}}^{2} \end{array}\right]⪰0.$$

In quantum mechanics, the physical variables $A_{i}$ correspond to Hermitian operators ${\hat{A}}_{i}$, the expected value becomes the quantum expectation value which can be theoretically calculated using the wavefunction, and so on, but the above definition is a general one. Moreover, Equation (1) straightforwardly entails known uncertainty relations within quantum mechanics: choosing $r=\langle [{\hat{A}}_{0},{\hat{A}}_{1}]\rangle /2$ (i.e., half of the commutator) leads to the Robertson uncertainty relation $\Delta_{{\hat{A}}_{1}}^{2}\Delta_{{\hat{A}}_{0}}^{2}\geq {\left|\langle \left[{\hat{A}}_{0},{\hat{A}}_{1}\right]\rangle \right|}^{2}/4$, while choosing $r=\langle {\hat{A}}_{1}{\hat{A}}_{0}\rangle -\langle {\hat{A}}_{1}\rangle \langle {\hat{A}}_{0}\rangle$ (i.e., the covariance) leads to the Schrödinger uncertainty relation

(2) $$\Delta_{{\hat{A}}_{0}}^{2}\Delta_{{\hat{A}}_{1}}^{2}\geq {\left(\frac{1}{2}\langle \left\{{\hat{A}}_{0},{\hat{A}}_{1}\right\}\rangle -\langle {\hat{A}}_{0}\rangle \langle {\hat{A}}_{1}\rangle \right)}^{2}+{\left(\frac{1}{2i}\langle \left[{\hat{A}}_{0},{\hat{A}}_{1}\right]\rangle \right)}^{2}.$$

The former choice of *r* is the reason we will identify below quantum uncertainty with non-zero commutation relations, while later we will use the latter choice to connect more generally the notion of uncertainty (or indeterminism) with local correlations (and often we would divide the covariance by the product of standard deviations resulting in the Pearson correlation coefficient).

It should be noted that, here, we chose what we believe to be the simplest notion of uncertainty in quantum mechanics and more general theories, i.e., a bound on the product of variances, but other inequalities exist involving the sum of the variances [2,3,4] or the entropy (entropic uncertainty relations) [5,6,7,8,9,10,11,12].

In what follows, we will begin with simple, yet deep, manifestations of quantum uncertainty, and then we shall discuss more recent ones, including a few novel implications of uncertainty for various quantum phenomena, and mainly nonlocality [13,14,15,16]. The latter results may be found in Section 4.

## 2. Immediate Observations

A quantum particle goes through a double slit. At first, the inability to retrieve both which-path information and interference may seem glooming, but in fact the uncertain position of the particle within the double slit is utterly necessary for observing later the interference pattern. Coherent superposition and all its fundamental consequences, like the wave-particle duality [17], as well as its practical consequences, e.g., quantum key distribution [18,19] and quantum computation [20,21], result from uncertainty. For instance, the Hadamard gate $H_{1}$, so valuable in quantum computation, is meaningful because it does not commute with the projectors |0〉〈0| and |1〉〈1| on the computational basis elements. Delayed choice [22] or eraser variants thereof [23,24,25] similarly depend on the uncertainty for reviving the which-path information.

The above relation between interference and uncertainty has clearly motivated the treatment of quantum particles as waves, which is familiar from the Schrödinger picture, but was also shown to take place within the particle-based, operator-oriented Heisenberg picture [26,27,28].

Obviously, the Heisenberg equation for the time evolution of an operator *A*, i.e.,

(3) $$\frac{d}{dt}A\left(t\right)=\frac{i}{\hbar }\left[H,A\left(t\right)\right]+\left(\frac{\partial A}{\partial t}\right),$$

depends on the commutator of *A* and the Hamiltonain *H*. The commutator, in return, determines the extent of uncertainty via, e.g., the Schrödinger-Robertson uncertainty relations, along with the anti-commutator.

Similarly, the von Neumann equation

(4) $$\frac{\partial }{\partial t}\rho =-\frac{i}{\hbar }\left[H,\rho \right],$$

crucially involves the commutator of the Hamiltonian with the density matrix ρ. The same is true of course for the dynamics of open quantum systems, relativistic particles and fields, etc., in which equations of motion, too, depend on non-trivial commutation relations.

Still within the Heisenberg picture, qualitative uncertainty relations were shown in Ref. [28] to prevent nonlocal equations of motion of modular operators from violating causality. We have demonstrated that the dynamics of quantum operators is markedly different from the classical dynamics of their classical counterpart in that certain quantities depend on potentials acting elsewhere. That could have led to violations of causality, but quantum uncertainty was able to mask this effect [28].

In addition to coherent superposition and quantum equations of motion, uncertainty relations are also responsible for quantization (see, e.g., Ref. [29]). It is customary to begin the quantization of the classical harmonic oscillator with the quantum commutation relation $[a,a^{†}]=1$ of the creation and annihilation operators resulting from the canonical commutation relation [x,p]=iℏ between the position and momentum operators. The latter commutation relation is arguably the most fundamental difference between classical and quantum mechanics. And indeed, the same methodology carries over to the quantization of fields, most notably the electromagnetic ones. Hence, the algebraic structure of quantum mechanics depending on the commutator, rather than the Poisson bracket, makes it utterly different. For a more thorough mathematical analysis see [30]. Despite the above, we note the smooth transition between quantum and classical mechanics through the group contraction ℏ→0.

## 3. Additional Observations

In many cases, uncertainty acts as a sea to which little droplets can be poured without being noticed [31,32]. This, for example, is the case with weak measurements [33]: A pointer is coupled to the system of interest in order to measure it. If the coupling is weak enough, or short enough in duration, so that the eventual shift of the pointer is much smaller than its uncertainty, then the measurement can be considered weak. Uncertainty can also mask the dependency of the weak value of any operator *A*, i.e., $A_{w}=\frac{\langle \phi |A|\psi \rangle }{\langle \phi |\psi \rangle }$, on the postselected state |ϕ〉, even when the weak value is anomalous [34,35,36,37,38,39] (i.e., lying outside the spectrum of *A*). This shielding of the future boundary condition is crucial of course for preventing causality violations.

This idea of “asymmetric” interaction (in terms of the involved uncertainties) is captured by “quantum oblivion” [31], where one particle seems to contain a record of a past interaction with another particle which remains oblivious of that. Quantum oblivion was shown to underlie interaction-free measurement [40], the quantum Zeno effect [41], the Aharonov-Bohm effect [42], and other quantum peculiarities.

We have also shown that, in the case of quantum hidden variables, lack of knowledge prevents them from signaling in time [43]; hence, they must remain unknown. Here, too, it was clear that full knowledge is not a bliss; better if certain physical variables remain hidden forever [44].

Although less obvious, the spin-statistics theorem can be also derived solely based on commutation and anti-commutation relations [45].

We shall now turn to the main topic of this paper, namely examining more closely the relations between nonlocality and uncertainty even beyond quantum mechanics. However, we hope that the above concise examples have helped clarifying the more general depth and significance of uncertainty in quantum mechanics.

## 4. Uncertainty and Nonlocality: A Quantum Intimacy

In the 1980s, Shimony [46,47] and Aharonov [48] conjectured independently that quantum mechanics is as nonlocal as it is, without violating causality, thanks to the existence of uncertainty (Aharonov further claimed that uncertainty relations are vital for preserving temporal causality (see, e.g., [49,50])). Recently, we rigorously quantified this claim [1], with related approaches reported in Reference [51,52,53,54,55]. Some of the important features of our approach are: (1) A completely general framework, external to the Hilbert space structure of quantum mechanics, relying only on a well defined statistics of empirical outcomes. (2) The Robertson-Schrödinger uncertainty relations is found as a special case. (3) Not only the Tsirelson bound is found but also the Tsirelson-Landau-Masanes (TLM) bound [56,57,58], as well as new, hitherto unnoticed bounds. (4) It has already proved useful in deriving new outcomes [59,60,61].

Rather than the general construction outlined in Ref. [1], we wish to begin with an interesting relation between uncertainty and nonlocality within the Hilbert space structure. For this purpose, let us examine the standard Bell-Clauser-Horne-Shimony-Holt (Bell-CHSH) scenario with Alice measuring either $A_{0}$ or $A_{1}$ and Bob measuring either $B_{0}$ and $B_{1}$ (all variables are assumed to take ±1 values). In quantum mechanics, the above variables are described as operators, and we may define the CHSH operator as $S=A_{0}B_{0}+A_{0}B_{1}+A_{1}B_{0}-A_{1}B_{1}$. The variance of this operator must be non-negative, i.e., $\langle S^{2}\rangle \geq {\langle S\rangle }^{2}$. In other words:

(5) $$\left|\langle S\rangle \right|\leq \sqrt{4+2\langle A_{0}B_{0}A_{1}B_{0}-A_{0}B_{1}A_{1}B_{1}+A_{0}B_{0}A_{0}B_{1}-A_{1}B_{0}A_{1}B_{1}+A_{0}B_{0}A_{1}B_{1}-A_{0}B_{1}A_{1}B_{0}\rangle }.$$

This can be seen as a generalized Tsirelson bound. In quantum mechanics, the *A* operator commute with the *B* operators; hence, the first two differences are zero, and we are left with the following tighter-than-Tsirelson bound including only the last pair of expectation values:

(6) $$|\langle A_{0}B_{0}\rangle +\langle A_{0}B_{1}\rangle +\langle A_{1}B_{0}\rangle -\langle A_{1}B_{1}\rangle |\leq \sqrt{4+2(\langle A_{0}B_{0}A_{1}B_{1}\rangle -\langle A_{0}B_{1}A_{1}B_{0}\rangle )}.$$

From this inequality, we easily deduce that stronger-than-classical correlations are possible only when $B_{0}$ and $B_{1}$ do not commute. Maximal violation of the CHSH inequality corresponds to a maximal difference between $\langle A_{0}B_{0}A_{1}B_{1}\rangle$ and $\langle A_{0}B_{1}A_{1}B_{0}\rangle$, i.e., maximal uncertainty on Bob’s side (and the same claims are true of course for Alice’s operators $A_{0}$ and $A_{1}$). But there is another lesson to be learned here: The commutativity of Alice’s and Bob’s operator was the one countering the potentiality of reaching stronger-than-quantum correlations implied by Equation (5). Hence, local commutation relations discriminate classical from quantum correlations and nonlocal commutation relations discriminate quantum from strong-than-quantum correlations. This perfectly accords with the conclusions of [1,55].

It should be noted that the difference between $A_{0}B_{0}A_{1}B_{1}$ and $A_{0}B_{1}A_{1}B_{0}$ also lies at the heart of the Peres-Mermin magic square [62,63]. In that case, we see a discrepancy between $\sigma_{1x}\sigma_{2y}\sigma_{1y}\sigma_{2x}$ and $\sigma_{1x}\sigma_{2x}\sigma_{1y}\sigma_{2y}$. Nonlocality and contextuality, therefore, agree on the difference between products of some operators, but nonlocality is richer in that it also requires equality between other products of operators. This equality was shown in [1,55] to be crucial. For us, it stands for a subtle form of relativistic causality.

Importantly, the above reasoning does not depend on the Hilbert space structure. We have proposed in [1] a way to generalize this necessary locality of uncertainty relations in a way which would be applicable to any well-defined statistical theory. Below we revisit this approach.

### 4.1. Nonlocality and Uncertainty in General

Nonlocal correlations are fundamentally bounded by the parties’ uncertainty relations. This fact was shown to be a characteristic of any physical theory where nonlocal correlations are consistent with relativistic causality [1]. As an example, consider again the Bell-CHSH scenario. The Hilbert-space structure of quantum mechanics affords a statistical covariance matrix for the Alice-Bob observables, a generalization of the covariance matrix from probability theory. The quantum covariance matrix of $A_{0}$, $A_{1}$, and $B_{j}$ is expressed as

(7) $$C\left(A_{0},A_{1},B_{j}\right)=\left[\begin{array}{ccc} \langle B_{j}^{2}\rangle -{\langle B_{j}\rangle }^{2} & \langle A_{0}⊗B_{j}\rangle -\langle A_{0}\rangle \langle B_{j}\rangle & \langle A_{1}⊗B_{j}\rangle -\langle A_{1}\rangle \langle B_{j}\rangle \\ \langle A_{0}⊗B_{j}\rangle -\langle A_{0}\rangle \langle B_{j}\rangle & \langle A_{0}^{2}\rangle -{\langle A_{0}\rangle }^{2} & \langle A_{0}A_{1}\rangle -\langle A_{0}\rangle \langle A_{1}\rangle \\ \langle A_{1}⊗B_{j}\rangle -\langle A_{1}\rangle \langle B_{j}\rangle & \langle A_{1}A_{0}\rangle -\langle A_{1}\rangle \langle A_{0}\rangle & \langle A_{1}^{2}\rangle -{\langle A_{1}\rangle }^{2} \end{array}\right],$$

which is a self-adjoint positive semi-definite matrix. It is sometimes convenient to normalize the rows and columns of $C(A_{0},A_{1},B_{j})$ by the respective standard deviations of $A_{i}$ and $B_{j}$ so as to obtain the quantum analog of a correlation matrix,

(8) $$\mathrm{Corr}\left(A_{0},A_{1},B_{j}\right)=\left[\begin{array}{ccc} 1 & \varrho (A_{0}⊗1,1⊗B_{j}) & \varrho (A_{1}⊗1,1⊗B_{j})\\ \varrho (A_{0}⊗1,1⊗B_{j}) & 1 & \varrho (A_{0},A_{1})\\ \varrho (A_{1}⊗1,1⊗B_{j}) & \varrho (A_{1},A_{0}) & 1 \end{array}\right],$$

where

(9) $$\varrho \left(X,Y\right)=\frac{\langle XY\rangle -\langle X\rangle \langle Y\rangle }{\sqrt{\langle X^{2}\rangle -{\langle X\rangle }^{2}}\sqrt{\langle Y^{2}\rangle -{\langle Y\rangle }^{2}}}$$

is the quantum counterpart of the Pearson correlation. This correlation matrix is similarly positive semi-definite, namely it satisfies

(10) $$\mathrm{Corr}\left(A_{0},A_{1}\right)⪰\left[\begin{array}{c} \varrho (A_{0}⊗1,1⊗B_{j})\\ \varrho (A_{1}⊗1,1⊗B_{j}) \end{array}\right]\left[\begin{array}{cc} \varrho (A_{0}⊗1,1⊗B_{j}) & \varrho (A_{1}⊗1,1⊗B_{j}) \end{array}\right]$$

by the Schur complement condition for positive semi-definiteness. The correlation matrix on the left, $\mathrm{Corr}(A_{0},A_{1})$, is the 2×2 lower submatrix in $\mathrm{Corr}(A_{0},A_{1},B_{j})$. Its non-negativity is equivalent to the Schrodinger-Robertson uncertainty relation, which follows from the non-negativity of its determinant, $1-|\varrho (A_{0},A_{1}){|}^{2}\geq 0$. Here, however, it may be recognized that Alice’s uncertainty relations become tighter due to the presence of Bob – the (matrix) lower bound on $\mathrm{Corr}(A_{0},A_{1})$ is no longer zero. Alternatively, the above matrix inequality can be viewed as Alice’s local bound ($\mathrm{Corr}(A_{0},A_{1})$) on the nonlocal Alice-Bob correlations ($\varrho (A_{i}⊗1,1⊗B_{j})$). Clearly, the roles of Alice and Bob can be switched to get a similar bound on nonlocal correlations, this time with Bob’s local uncertainty relations, $\mathrm{Corr}(B_{0},B_{1})$.

We have shown in Ref. [1] that similar matrix inequalities lead to known, as well as new, characterizations of the set of bipartite quantum correlations. Apart from the well-known Tsirelson’s bound, other characterizations may involve nonlinear functions of the underlying correlations. To get a more intuitive expression relating the Bell-CHSH parameter and the local uncertainties of Alice and Bob, let us assume the correlations are isotropic, $\varrho \left(A_{i}⊗1,1⊗B_{j}\right)={\left(-1\right)}^{ij}c$, for some c∈[−1,1]. The Bell-CHSH parameter in this case is, $S=\sum_{i,j\in \{0,1\}}{\left(-1\right)}^{ij}\varrho \left(A_{i}⊗1,1⊗B_{j}\right)=4c$. Plugging these correlations into the matrix inequality above reads

(11) $$\mathrm{Corr}\left(A_{0},A_{1}\right)⪰c^{2}\left[\begin{array}{cc} 1 & {\left(-1\right)}^{j}\\ {\left(-1\right)}^{j} & 1 \end{array}\right],$$

which is equivalent to the non-negativity of the determinant of the matrix obtained by subtracting the right side from the left side above,

(12) $$2c^{2}+{\left|\varrho (A_{0},A_{1})\right|}^{2}-{\left(-1\right)}^{j}c^{2}\left(\varrho \left(A_{0},A_{1}\right)+\varrho \left(A_{1},A_{0}\right)\right)\leq 1.$$

Adding together the inequalities for j=0,1 and recalling that c=S/4 leads to

(13) $${\left(\frac{S}{2\sqrt{2}}\right)}^{2}\leq 1-{\left|\varrho (A_{0},A_{1})\right|}^{2}=\det\left(\mathrm{Corr}(A_{0},A_{1})\right)$$

and, by switching the roles of Alice and Bob,

(14) $${\left(\frac{S}{2\sqrt{2}}\right)}^{2}\leq 1-{\left|\varrho (B_{0},B_{1})\right|}^{2}=\det\left(\mathrm{Corr}(B_{0},B_{1})\right).$$

These show that quantum nonlocality, as measured by the Bell-CHSH parameter, is bounded by Alice’s and Bob’s local uncertainties, as quantified by determinants of the respective (local) correlation matrices. It can also be noticed that Tsirelson’s bound in that case, the $2\sqrt{2}$, is attained for the maximum uncertainty on both sides, when $\det\left(\mathrm{Corr}(A_{0},A_{1})\right)=\det\left(\mathrm{Corr}(B_{0},B_{1})\right)=1$.

Similar relations apply to multiple-input-multiple-output, multipartite scenarios [1,55]. Moreover, this approach can be extended through the use of “complex correlations” to non-Hermitian, signaling operators [60] and to continuous variables [61]. Finally, our approach has given rise to multiplicative Bell inequalities and their Tsirelson bounds [59], as well as several other results currently underway. For some related (and very interesting) analyses see [51,53,54].

In all these cases, we have seen that, although uncertainty relations enable correlations beyond quantum mechanics, local consistency (i.e., the independence of uncertainty relations and local correlations on the choices of remote parties) constrains the nonlocal correlations to lie within the boundaries of quantum mechanics.

### 4.2. Uncertainty as an Axiom

Violations of Bell inequalities are experimental fact and hence make part of the predictions of any physical theory which may someday replace quantum mechanics. On the other hand, violations of relativistic causality have never been witnessed and are believed to lead to grievous paradoxes. Nevertheless, taking nonlocality as one of the axioms of a physical theory and relativistic causality as the other has proved futile in characterizing the set of quantum correlations—relativistic causality does not limit the strength of nonlocality whatsoever [64]. Partial characterizations of this set have been derived using reasonable, though not always physical, arguments [51,65,66,67,68,69,70,71,72].

However, it was recently shown that, once uncertainty relations, broadly understood, are taken as a starting point, relativistic causality, manifested by the locality of uncertainty relations, completely characterizes the set of quantum correlations in a bipartite binary measurement setting [1]. Uncertainty relations in the sense used here refer to the existence of an empirical covariance matrix, which is far less than assuming the Hilbert-space structure of quantum mechanics. Such a covariance may be written for any number of experimenters, with any number of measurement devices, and for both discrete and continuous variables. Further assuming locality of uncertainty relations—that experimenters cannot tamper with the uncertainty relations of their peers—restricts the set of nonlocal correlations.

### 4.3. Uncertainty, Randomness, and Nonlocality

The amount of nonlocality present in a multipartite quantum mechanical system is related both to the local uncertainty relations and to the predictability of measurement outcomes. For ±1-valued observables, *A* and *B*, we may take 〈A〉 and 〈B〉 as indicators of the randomness inherent to Alice’s and Bob’s measurements—both expected values vanish for completely random outcomes. Assume for simplicity that the expected values of Alice/Bob observables are the same. We can now prove the following bound on the Bell-CHSH parameter,

(15) $$\left|S\right|\leq 2+\left[2\sqrt{2(1-\eta^{2})}-2\right]\sqrt{\left(1-{\langle A\rangle }^{2}\right){(1-\langle B\rangle }^{2}},$$

where η equals either $|\varrho (A_{0},A_{1})|$ or $|\varrho (B_{0},B_{1})|$, with the larger providing a tighter bound. This shows that both quantities, the local uncertainty $\sqrt{1-\eta^{2}}$, and measurement predictability, as quantified by the right-hand side term in the square root, dictate the amount of nonlocality. We have previously shown [55] that this relation can be alternatively quantified via the Tsallis entropy [73] of parameter q=1/2. Moreover, the above relation between local and nonlocal correlations, as well as the aforementioned ones, can be tested in the lab using sequential weak measurements [74] performed on each of the photons within a Bell test setup (also see [55,60]).

It is worth noting that other manifestations of quantum nonlocality, such as discord [75,76,77] and reactivity [78], are known to depend on local uncertainty relations [78,79,80].

## 5. Discussion

We have briefly discussed the immense explanatory power of local uncertainty relations in quantum mechanics, especially the quantitative characterization of quantum nonlocality. We leave the following as open questions:

1. Does any local uncertainty relation (including, e.g., entropic uncertainty relations) correspond to a meaningful bound on nonlocal correlations?
2. Is there a finite pathway for deriving *tight* bounds on quantum correlations?
3. How would dynamical nonlocality seem in theories beyond quantum mechanics?
4. Are there quantum phenomena which cannot be traced back to quantum uncertainty?
5. Are uncertainty and causality the fundamental axioms to begin with (similarly to our analysis in Ref. [1]), or is there a conceptually superior set of axioms?
